# Imaging of auriculotemporal nerve perineural spread

**DOI:** 10.3332/ecancer.2013.374

**Published:** 2013-11-19

**Authors:** Michael Chan, Adam A. Dmytriw, Eric Bartlett, Eugene Yu

**Affiliations:** 1 Joint Department of Medical Imaging, University Health Network, 101 College St, Toronto, ON, M5G 1L7, Canada; 2 Department of Medical Imaging, Princess Margaret Hospital, 610 University Ave, Toronto, ON, M5T 2M9, Canada

**Keywords:** perineural spread, head and neck malignancy, MRI, CT

## Abstract

**Importance:**

Adenoid cystic carcinomas (ACCs) are relatively rare tumours, notorious for wide local infiltration and perineural spread. Perineural extension commonly occurs along branches of the trigeminal and facial nerves, and its presence represents a poor prognostic factor with implications for treatment approach.

**Observations:**

We report the case of a 61-year-old female presenting with worsening left facial numbness and weakness. On magnetic resonance imaging, the patient was found to have perineural spread of a left parotid tumour along the auriculotemporal nerve. There was involvement of the V2 and V3 branches of the trigeminal nerve. An ultrasound-guided biopsy of the mass demonstrated ACC.

**Conclusions and relevance:**

The auriculotemporal nerve may serve as a route for tumour spread, particularly in the setting of head and neck malignancy. Moreover, this particular suspicion should be raised when patients with known malignancy experience concomitant trigeminal (V) and facial (VII) nerve dysfunctions.

## Introduction

Adenoid cystic carcinoma (ACC) is a slow-growing but aggressive malignant tumour with a propensity for local recurrence and late distant metastasis. Although it is relatively rare, constituting only 1% of all malignant oral and maxillofacial tumours, ACC comprises approximately 15% of parotid gland malignancies [1, 2]. It is characterised by wide local infiltration and is well known for its tendency for perineural spread. Epidemiologically, ACC exhibits a slight female predilection and has a peak incidence in the fifth and sixth decades of life [2]. Clinically, patients typically present with signs and symptoms related to local tissue invasion and perineural spread [3]. The most commonly involved nerves are the facial nerve, as well as the maxillary (V2) and mandibular (V3) divisions of the trigeminal nerve [4, 5]. It is thought that pre-existing connections between the facial and the trigeminal nerves, including the auriculotemporal nerve, aid in the perineural dissemination of tumour between these two nerves [5]. In this report, we present a case of ACC arising in the parotid gland with extension along the auriculotemporal nerve.

## Case report

**Figure 1.**
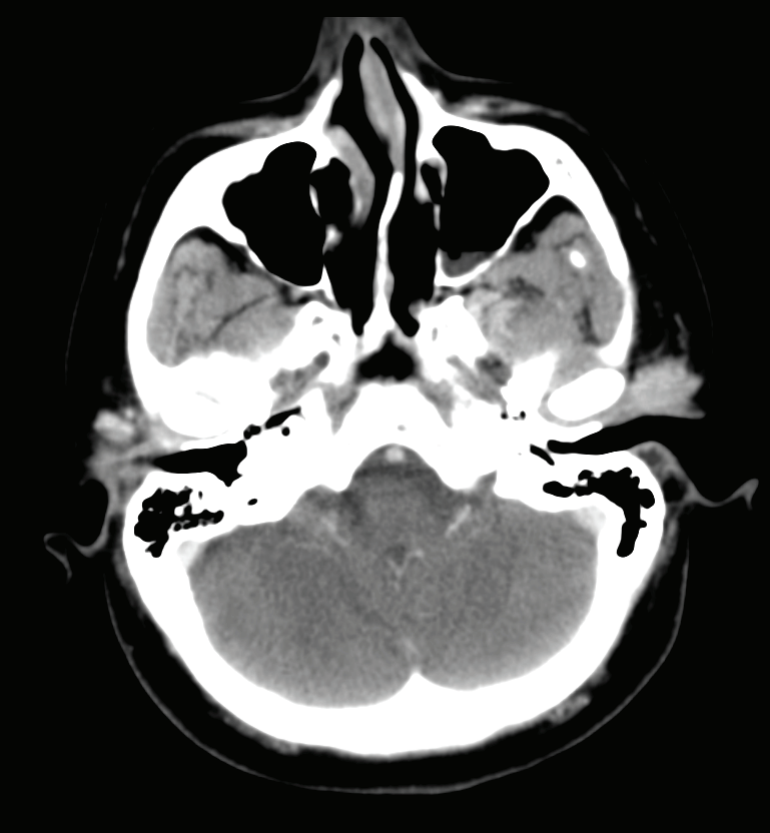
A contrast-enhanced axial CT image shows an enhancing, lobulated mass in the left preauricular region.

A 61-year-old woman presented four years prior with a painless mass involving the left parotid region. The patient’s medical history was non-contributory. There were no other neurologic signs or symptoms and no history of malignancy. A computed tomography (CT) scan of her head performed four years previously demonstrated an ill-defined lesion in the superficial left parotid gland that corresponded to a palpable preauricular mass (Figure 1). The clinician recommended that this lesion be followed conservatively with observation. However, the lesion gradually increased in size over time. The patient then developed increasing numbness in the left mandibular region, weakness of the ipsilateral frontalis muscle, pain, and left-sided trismus. An ultrasound-guided core biopsy was performed, which demonstrated an intermediate-grade (2/3) ACC. She was then referred to our tertiary oncology centre. Physical examination revealed a firm, tender left parotid mass as well as left Level II adenopathy. There was numbness involving the left V2 and V3 distribution and slight left facial nerve weakness.

**Figure 2 A–C.**
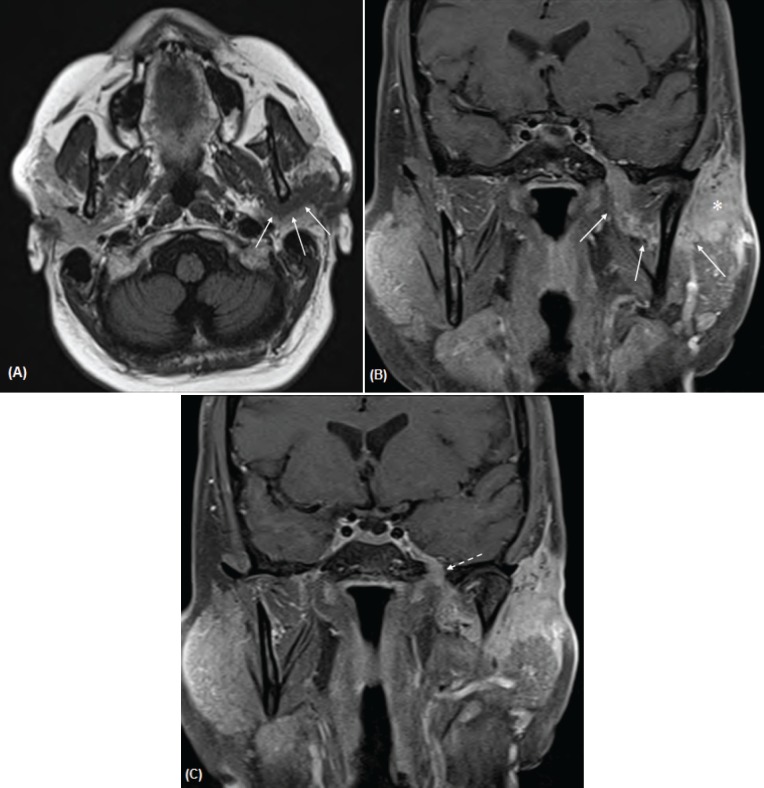
Axial T1-weighted image (A) and post-contrast coronal T1-weighted images with fat saturation (B, C) demonstrates a curvilinear band of enhancing tumour (arrows in A, B) that extends from the left parotid mass (asterisk) and tracks behind the mandibular ramus and joins with the V3 nerve in the masticator space. There is contiguous extension along V3 superiorly through a widened foramen ovale (dashed arrow in C) and asymmetric thickening and enhancement of the left cavernous sinus.

A magnetic resonance imaging (MRI) examination was performed (Figure 2). This demonstrated a lobulated mass measuring 2.0 × 2.2 cm within the superficial lobe of the left parotid gland with extension to the overlying capsule and thickening of the overlying skin. Thick curvilinear enhancing tissue was noted to be extending from the parotid mass around the posterior ramus of the mandible and joining with the V3 trunk in the left masticator space that corresponds to the track of the auriculotemporal nerve. Contiguous thickening and enhancement along V3 superiorly through a widened foramen ovale was noted. There was also slight thickening in the adjacent inferior aspect of the left cavernous sinus. These findings were compatible with perineural tumour spread along the left auriculotemporal nerve with contiguous extension to involve the left V3.

The patient underwent a left parotidectomy with sacrifice of the facial nerve. Visible perineural tumour spread along the ATN was noted intraoperatively. Asural nerve graft from the ipsilateral hypoglossal nerve to the distal facial nerve was performed.This was followed by extended composite resection of the left mandibular condyle and ramus and the contents of the infratemporal fossa. Dissection was carried out up to the level of foramen ovale. Intraoperative frozen margins were positive despite best efforts.The mandible was reconstructed with a temporomandibular joint (TMJ) prosthesis, and a fasciocutaneous radial forearm flap was used for soft tissue coverage. A left tarsorrhaphy and static sling were also performed. A gold weight was also inserted into the left upper eyelid. Two of 32 nodes at left Level IIb were positive with extracapsular extension.

The patient then underwent postoperative radiation therapy consisting of 66 Gy in 33 fractions to the left skull base with coverage to the level of the left clavicle.She subsequently developed left external auditory canal stenosis that was treated with left meatoplasty and canaloplasty procedures. Following surgery (after 1.5 years), the patient has not demonstrated any overt evidence of disease recurrence. She demonstrates some minor movement in the left zygomatic region of the facial nerve on active closure.

## Discussion

ACC is one of the most common salivary gland malignancies. As shown in our patient, this is a locally invasive tumour with a notorious tendency toward perineural spread. In this case, the tumour was found to extend along the left auriculotemporal nerve with involvement of the mandibular (V3) division of the left trigeminal nerve.

The auriculotemporal nerve is one of the major known communications between the facial and trigeminal nerves. It arises as two nerve roots from V3 that course posteroinferiorly around the middle meningeal artery—the upper and lower roots extend lateral and medial to the artery, respectively—and coalesce into a short trunk medial to the TMJ and superior to the bifurcation of the external carotid artery into its temporal and maxillary branches. From there, it divides into multiple branches, including the anterior and posterior communicating rami that join the facial nerve within the parotid gland to supply sensory fibres to its zygomatic, buccal, and mandibular divisions [5]. Although not present in our patient, symptoms suggestive of auriculotemporal nerve involvement include periauricular pain and TMJ dysfunction and/or tenderness [5].

Imaging features of perineural tumour spread include thickening and abnormal enhancement along involved nerves, foraminal widening, and erosions due to tumour growth along the nerves, neuropathic atrophy of denervated muscles, as well as obliteration of perineural fat pads [4, 6]. For optimal detection of perineural invasion, high-resolution, fat-suppressed axial and coronal T1-weighted MRI with and without contrast is recommended [4, 6, 7]. Lesions typically exhibit abnormal contrast enhancement on post-contrast images, as well as abnormal signal hyperintensity on T2-weighted images [4]. However, negative radiologic studies may be obtained even in patients with tumour invasion [4, 6]. Therefore, it is important to correlate radiologic findings with clinical and pathologic data [8]. Perineural spread of other nerves in the head and neck exhibits the same behaviour, but auriculotemporal nerve involvement is of particular significance for its extension to both cranial nerves V and VII. Perineural spread can also occur in the setting of many other malignancies, including rhabdomyosarcoma, squamous cell carcinoma, and lymphoma, among others [6]. This spread can be identified on imaging both directly and indirectly. Direct findings include a large and/or enhancing nerve, and indirect findings include foramenal widening or destruction, loss of adjacent fat planes, and signs of denervation [9].

Histologically, ACC is composed of a mixture of epithelial and ductal cells and is assigned one of three histologic grades: Grade I, a well-differentiated tumour composed of tubular and cribriform patterns without solid components; Grade II, a tumour with a pure cribriform or mixture of patterns with solid growth pattern less than 30% of the tumour; and Grade III, a tumour with marked predominance of the solid pattern [10]. Higher grades are associated with larger size at presentation, higher recurrence rates, and higher mortality rates. Other prognostic factors include the tumour location, stage, presence or absence of surgical margins, and the anatomic structures involved (i.e., perineural spread) [2, 10].

Our patient underwent surgical resection of the mass with radical parotidectomy followed by adjuvant radiotherapy. Treatment of ACC is primarily surgical with or without radiation therapy. The goal of surgery is to obtain at least a 1-cm margin around the tumour [2]. However, the disease is extremely difficult to treat due to high rates of recurrence and metastases if the patient lives long enough, even despite radical surgery. The five-year survival rate after effective treatment is 75%, while long-term prognosis is poor, with a 10-year survival rate of 20% and 15-year survival rate of 10% [2]. Adjuvant radiotherapy combined with radical surgery has been shown to increase long-term survival.

The presence of perineural invasion represents a poor prognostic factor and has implications for the treatment approach, such as indicating the need for wider resection and expanded radiation field [4, 11]. Tumours with perineural spread have high recurrence rates and decreased survival. Patterns of perineural spread and knowledge of the anatomy of the cranial nerves may help in planning the appropriate treatment modalities.

As we demonstrate, the auriculotemporal nerve can serve as a route for tumour spread in the setting of head and neck malignancy. Moreover, this particular suspicion should be raised when patients with known malignancy experience concomitant trigeminal and facial nerve dysfunction. We report a case of an ACC of the parotid gland with perineural invasion of the auriculotemporal nerve and involvement of V2 and V3. High-resolution MRI with and without contrast is the imaging modality of choice for the detection of perineural tumour invasion. Due to its implications on prognosis and treatment, it is imperative to recognise the presence of perineural invasion.
